# Rectal foreign body of a cosmetic bottle treated successfully by transanal retrieval: A case report

**DOI:** 10.1097/MD.0000000000040651

**Published:** 2024-11-22

**Authors:** Congcong Liu, Yuantao Li

**Affiliations:** a First Clinical Medical College, The Afffliated Hospital of Shandong University of Traditional Chinese Medicine, Jinan, China; b First Clinical Medical College, Shandong University of Traditional Chinese Medicine, Jinan, China; c Department of Colorectal Surgery, The First Affiliated Hospital of Shandong First Medical University, Jinan, China.

**Keywords:** rectal foreign body, surgical treatment, transanal retrieval

## Abstract

**Rationale:**

Retained rectal foreign bodies (RFBs) are unusual clinical presentations whose management is challenging for emergency physicians owing to variations in the object types, anorectal anatomy, sacral curvature, insertion times, and local contamination. Here, we present the diagnosis and treatment in 1 case of retained rectal foreign body.

**Patient concerns:**

A 62-year-old male presented to the emergency department with a cosmetic bottle inserted into the rectum while bathing. He had difficulty defecating and denied any underlying psychiatric illness. Before coming to the hospital, the patient attempted to remove the cosmetic bottle with a screwdriver but failed, causing the cosmetic bottle to penetrate further into the intestinal lumen. The patient felt that the anal bulging was gradually worsening.

**Diagnoses:**

Investigations, a digital rectal examination, and coronal abdominal computed tomography revealed a foreign body stuck in the rectum.

**Interventions and outcomes:**

After lateral internal sphincterotomy, the cosmetic bottle’s plastic cap was pinched using a towel clamp and rotated slowly. The patient had an uneventful recovery period; the difficult defecation and anal bulging were relieved.

**Lessons:**

This case proves that lateral internal sphincterotomy can be performed to remove retained rectal foreign bodies if sufficient sphincter relaxation and anal dilatation cannot be achieved with proper anesthesia.

## 1. Introduction

Retained rectal foreign bodies (RFBs) are unusual clinical presentations in the emergency department, but the incidence has been increasing recently.^[1]^ Its management is challenging for emergency physicians because of variations in the object types, anorectal anatomy, sacral curvature, insertion times, and local contamination.^[2,3]^ We review the diagnosis and treatment in 1 case of RFB.

## 2. Case presentation

A 62-year-old male presented to the emergency department complaining of anal bulging for 2 days, difficulty defecating, and denial of any underlying psychiatric illness. The patient had a history of accidental slipping in the bathroom, leading to the insertion of a cosmetic bottle in the rectum while bathing 2 days prior. Prior to visiting the hospital, the patient attempted to remove the cosmetic bottle with a screwdriver but failed, causing the bottle to penetrate the intestinal lumen further. The patient felt that the anal bulging gradually worsened; he was in no other apparent distress. Physical examination showed consciousness, a body temperature of 97.5°F (36.4°C), a blood pressure of 162/91 mm Hg, a heart rate of 81/min, a respiratory rate of 20/min, and a percutaneous oxygen saturation of 99%. An abdominal examination was unremarkable, without clinical signs of peritonitis.

**Figure 1.**
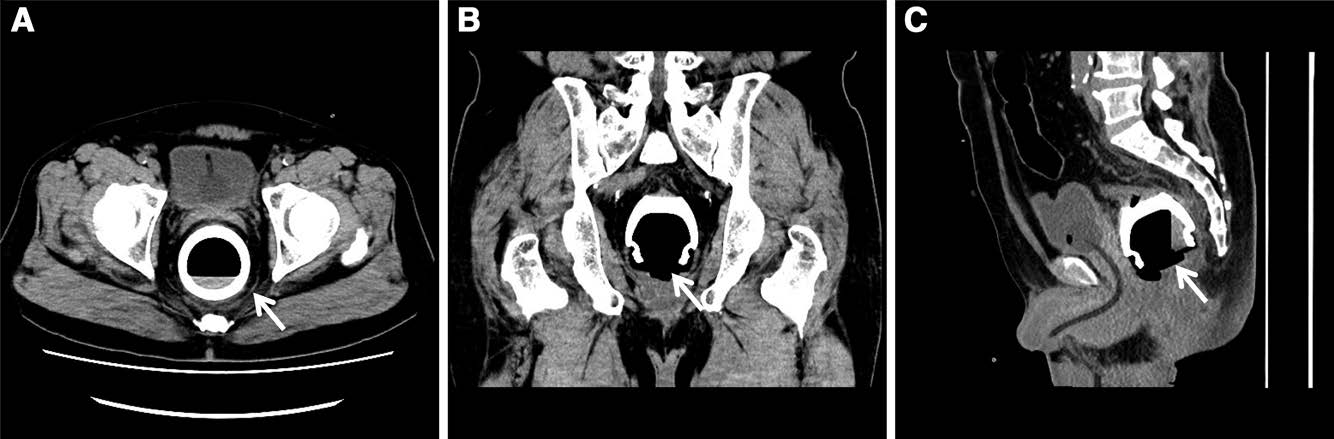
CT imaging shows a cup-shaped foreign body stuck in the rectal lumen (white arrow). (A) Axial view, (B) coronal view, (C) sagittal view. CT = computed tomography.

**Figure 2.**
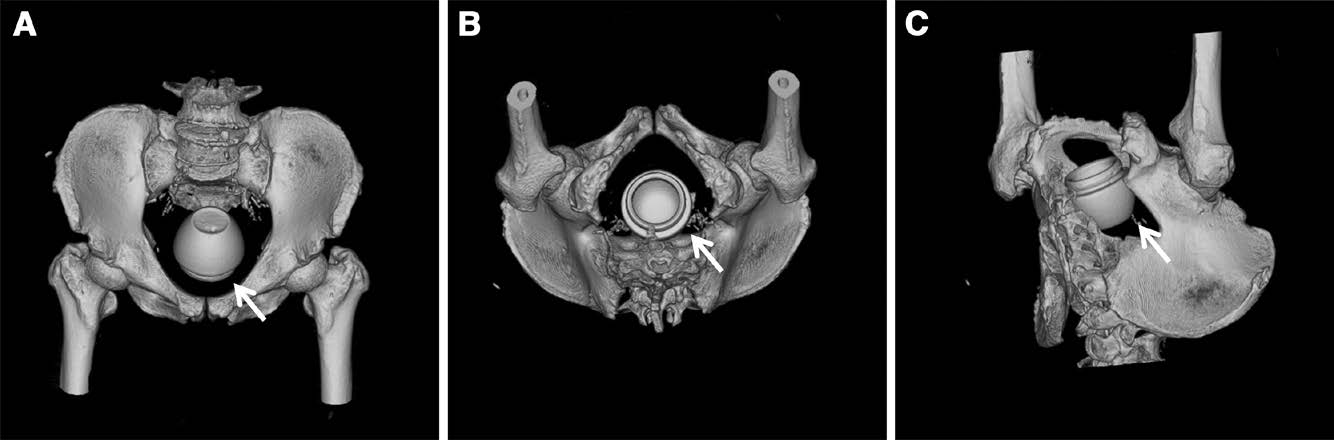
CT images (A–C) with the VRT show the 3D surface of the cup-shaped foreign body stuck in the rectal lumen (white arrow). 3D = three dimensional, CT = computed tomography, VRT = volume rendering technique.

The white blood cell count was 12.63 × 10^9^/L; other hemodynamic parameters were normal. On digital rectal examination, the base of the cosmetic bottle was palpated as an immovable solid object 5 to 6 cm proximal to the anus; a small amount of bloody mucus was found on the examining glove. Computed tomography with multiplanar reconstruction showed a dilated rectum, with a cup-shaped high-density shadow at the pubic symphysis and above, approximately 70 × 70 × 60 mm in size (Figs. 1 and 2), with no signs of intestinal perforation.

## 3. Treatment and outcome

**Figure 3.**
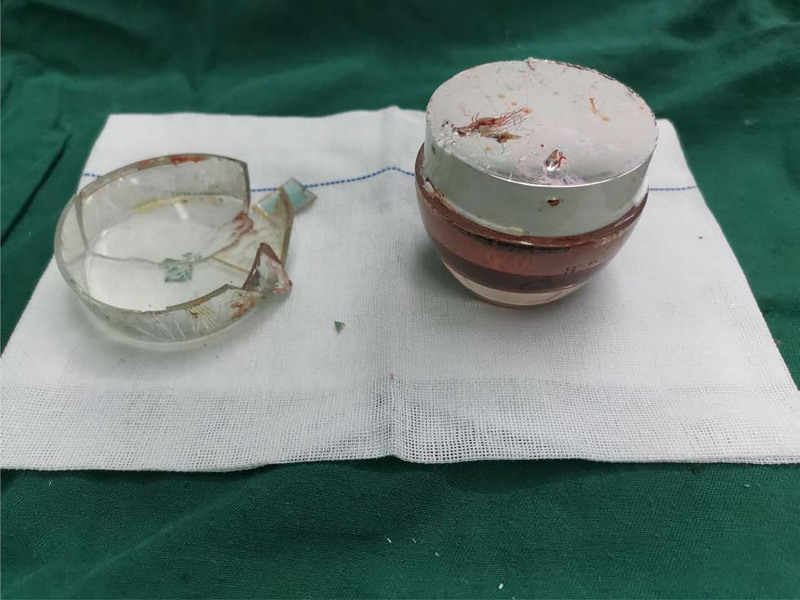
FB after removal. FB = foreign body.

The patient was hospitalized for surgical treatment. The patient was placed in the lithotomy position in the operating room under spinal anesthesia. The assistant applied gentle suprapubic pressure to help move the object caudally. We attempted to remove the cosmetic bottle directly through the anus with oval forceps and forceps. However, we failed because of its round, smooth shape, the vacuum effect on the colonic wall, and the limited dilatation capacity of the anus. Subsequently, the plastic cap distal to the cosmetic bottle was removed by crushing the bottle. A lateral internal sphincterotomy (LIS) was performed. A small incision was made on the right side of the anal skin to expose the internal sphincter muscle fibers. The internal anal sphincter muscle was lifted, and it was divided using thermal cautery. Cutting the muscle relaxed the pressure in the anus, allowing the cosmetic bottle’s plastic cap to be pinched using a towel clamp and rotated slowly. After removing the foreign body (FB) from the rectum (Fig. 3), anoscopy revealed that the rectal wall’s mucosa had localized redness, congestion, and scattered superficial ulcers due to prolonged compression. An absorbable suture was used to fold the internal sphincter.

No severe complications occurred after the surgery. The patient was discharged on the second postoperative day. The symptoms of difficult defecation and anal bulging were relieved, and sphincter function was good. The patient did not return to the outpatient clinic for a follow-up examination.

## 4. Discussion

RFBs are relatively rare in emergency departments, but their incidence has increased recently.^[1]^ Usually, FBs are inserted purposely for relief from constipation, assault, assisted laxation, self-gratification associated with anal eroticism, or psychiatric reasons.^[4,5]^ Regardless of the purpose, inserting FBs into the anus is considered a taboo behavior. Therefore, most patients are reluctant to report an FB, and they attempt to remove it independently, delaying presentation. Objects inserted into the anus include sex toys, wine bottles, eggplants, pipes, radishes, light bulbs, and pesticide bottles; however, a cosmetic container has rarely been reported.^[3,6,7]^ Patients may present with anal distension, constipation, bleeding, intestinal obstruction, abdominal pain, abdominal distention, and peritonitis. Sometimes, the patients may not be honest in disclosing details regarding a self-inserted rectal FB but may provide an unrealistic cause since it may be embarrassing. Even without an accurate history, RFBs are diagnosed readily via digital rectal examination or imaging studies, such as plain radiography and computed tomography. However, managing RFBs requires an individualized approach based on the nature, size, shape, and location of the impacted affected FB and the degree of FB-induced rectal injury, which ranges from mucosal injury to colorectal perforation.

Extraction techniques include transanal, endoscopic, and laparotomy with repair of complications.^[8]^ In the absence of emergencies, such as bowel perforation and peritonitis, the preferred treatment strategy is to minimize trauma by removing the FB through an anal approach.

Purely direct transanal removal may be difficult because of the FB’s shape, anorectal anatomy, sacral curvature, and anal sphincter spasm. If sufficient sphincter relaxation and anal dilatation cannot be obtained with proper anesthesia, LIS can be performed to remove RFBs. LIS is commonly indicated for conditions such as chronic anal fissures, recurrent fissures, hypertonicity of the internal anal sphincter, and severe anal pain; however, its application in this case was aimed at facilitating removal of the rectal FB. The LIS helped reduce the internal sphincter’s tone, providing greater space and flexibility, which are crucial for safely extracting the object. This approach minimizes the risk of further trauma to the rectal mucosa and the surrounding tissues.

Larger or elongated objects pose distinct challenges and may necessitate different approaches. For example, Ho and Su^[2]^ documented a case of a 23 cm pesticide bottle retained in the rectum, emphasizing the complexity involved in managing such a large FB; the object’s length and rigidity may impede simple transanal removal. When transanal retrieval is unsuccessful, laparotomy may be required to extract the object safely without causing additional injuries.

## 5. Conclusion

RFBs are infrequent in the emergency department; the objects include several things. However, a cosmetic container has rarely been reported. The choice of treatment strategy is challenging for emergency physicians because of patients’ clinical conditions and variation among object types. LIS enables removing RFBs in a minimally traumatic way when sufficient sphincter relaxation and anal dilatation are unobtainable with proper anesthesia. In addition, if a patient presents with an associated emergency, such as bowel perforation or peritonitis, a more invasive abdominal route may be promptly needed, such as laparotomy or laparoscopy.

## Acknowledgments

The authors express our gratitude to the participants for their valuable contributions and consent to participate.

## Author contributions

**Funding acquisition:** Congcong Liu.

**Methodology:** Congcong Liu.

**Writing—original draft:** Congcong Liu.

**Data curation:** Yuantao Li.

**Supervision:** Yuantao Li.

**Writing—review & editing:** Yuantao Li.
